# A promising biodegradable magnesium alloy suitable for clinical vascular stent application

**DOI:** 10.1038/srep46343

**Published:** 2017-04-11

**Authors:** Lin Mao, Li shen, Jiahui Chen, Xiaobo Zhang, Minsuk Kwak, Yu Wu, Rong Fan, Lei Zhang, Jia Pei, Guangyin Yuan, Chengli Song, Junbo Ge, Wenjiang Ding

**Affiliations:** 1 Shanghai Institute for Minimally Invasive Therapy, School of Medical Instrument and Food Engineering, University of Shanghai for Science and Technology, Shanghai, 200093, China; 2 National Engineering Research Center of Light Alloys Net Forming and State Key Laboratory of Metal Matrix Composite, Shanghai Jiao Tong University, Shanghai, 200240, China; 3 Department of Biomedical Engineering, Yale University, New Haven, CT 06511, USA; 4 Shanghai Institute of Cardiovascular Diseases, Department of Cardiology, Zhongshan Hospital, Fudan University, Shanghai, 200032, China; 5 School of Materials Science and Engineering, Nanjing Institute of Technology, Nanjing, 211167, China

## Abstract

We report a Mg alloy Mg-2.2Nd-0.1Zn-0.4Zr (wt.%, denoted as JDBM-2) showing great potential in clinical vascular stent application by integrating the advantages of traditional medical stainless steel and polymer. This alloy exhibits high yield strength and elongation of 276 ± 6 MPa and 34.3 ± 3.4% respectively. The JDBM-2 with a stable degradation surface results in a highly homogeneous degradation mechanism and long-term structural and mechanical durability. *In vitro* cytotoxicity test of the Mg extract via human vascular endothelial cells (HUVECs) indicates that the corrosion products are well tolerated by the tested cells and potentially negligible toxic effect on arterial vessel walls. This alloy also exhibits compromised foreign body response (FBR) determined by human peripheral blood derived macrophage adhesion, foreign body giant cell (FBGC) formation and inflammatory cytokine and chemokine secretion. Finally, vascular stents manufactured from the JDBM-2 were implanted into rabbits for long-term evaluation. The results confirm excellent tissue compatibility and up to 6-month structural and mechanical integrity of the stent *in vivo*. Thus, the JDBM-2 stent with up to 6-month structural and mechanical integrity and excellent tissue compatibility represents a major breakthrough in this field and a promising alternative to traditional medical stainless steel and polymer for the clinical application.

Coronary heart disease (CHD), the number one killer of both men and women worldwide, counts for 34 and 41% of all death in the United States^1^ and in China^2^, respectively. Since the idea of placing stents in blood vessels was first proposed by Dotter *et al*. in 1964^3^, coronary stenting has evolved into the most common catheter-based treatment of coronary heart disease due to its lower restenosis rate than percutaneous transluminal coronary angioplasty (PTCA)^4^.

In the past decades, cardiovascular stents have been manufactured using two material classes: metals and polymers. Of these two, polymers for load-bearing application are relatively weak and less rigid compared with metals. Moreover, polymer stents are also associated with an increased risk of late stent thrombosis (ST)^5^. The conventional metallic implants based on stainless steel^6^,^7^,^8^, Co-Cr alloy^9^,^10^,^11^, Ni-Ti alloy^12^,^13^,^14^ and Ta^15^ are the commonly used materials for coronary artery reconstruction because of their high mechanical properties and excellent corrosion resistance. However, the long-term host foreign body response (FBR) to permanent stent implantation often results in restenosis or lumen encroachment, brought about by the recruitment of inflammatory and smooth muscle cells to the intima with subsequent proliferation and extracellular matrix (ECM) deposition^16^,^17^. Hence, metallic materials, which can be absorbed and incorporated into the tissues, appear to provide an approach to avoid the long-term endothelial dysfunction and foreign body reaction. Iron and Mg alloys are possible candidates for use in biomedical implants. Whereas the corrosion period of iron is too long^18^, Mg-based implants appear to offer appropriate degradation periods being closer to the clinical requirements^19^. Moreover, Mg alloys show excellent anti-platelet deposition and low thrombogenicity, which makes it particularly promising for cardiovascular intervention^20^,^21^. More importantly, Mg is one of the trace elements existing in the human body, the corrosion product of Mg alloy generated by the electrochemical reaction Mg + 2H_2_O → Mg (OH)_2_ + H_2_ can be absorbed or excreted by the surrounding tissues and metabolic system^22^. Previous studies have showed great promising of Mg alloys for biomedical application since the first Mg stent was implanted into a preterm baby in 2004 by Zartner *et al*.^19^,^23^,^24^.

Despite recent advances in the improvement of corrosion properties via a variety of technical methods, Mg alloys for the clinical application of biodegradable cardiovascular stent is still challenged by the short-term support of less than 6-month durability due to the rapid galvanic-corrosion, which is undesirable to cause complete failure of the medical device before the tissue has healed completely. Here, we report a promising Mg alloy JDBM-2 with great potential by integrating the advantages of medical stainless steel and biodegradable polymer for vascular stent application. The cardiovascular stent manufactured by this Mg alloy with up to 6-month structural and mechanical integrity and excellent tissue compatibility achieved through controllable homogeneous degradation mechanism represents a major advancement in the evolution of stent and also opens opportunities to improve the long-term clinical outcome of percutaneous coronary intervention.

## Results

### Microstructures and Mechanical properties

**Figure 1:**
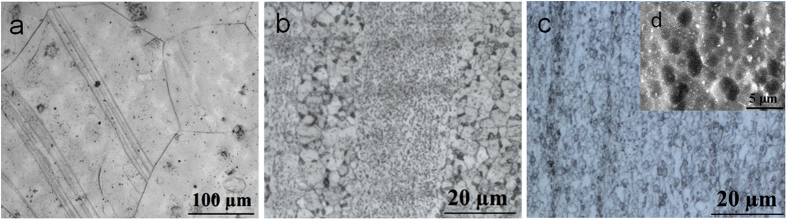
Microstructures of the JDBM-2 alloy in (**a**) T4, (**b**) once extrusion and (**c**) double extrusion conditions. (**d**) The inset in (**c**) showing SEM image of double extruded JDBM-2 dispersed with nano particles in grain interiors and boundaries.

Figure 1a–c shows the microstructures of the JDBM-2 in solution treatment (T4), once extrusion and double extrusion conditions. The microstructure of the JDBM-2 after double extrusion becomes much finer and more homogeneous than those in T4 and once extrusion conditions due to complete dynamic recrystallization in hot processing. Fine dispersed nano particles (precipitated phases) which contain 3.0 wt% Nd and 1.6 wt% Zr precipitates in grain interiors and boundaries of the JDBM-2 after double extrusion (Fig. 1d, Figure 1s).

**Figure 2:**
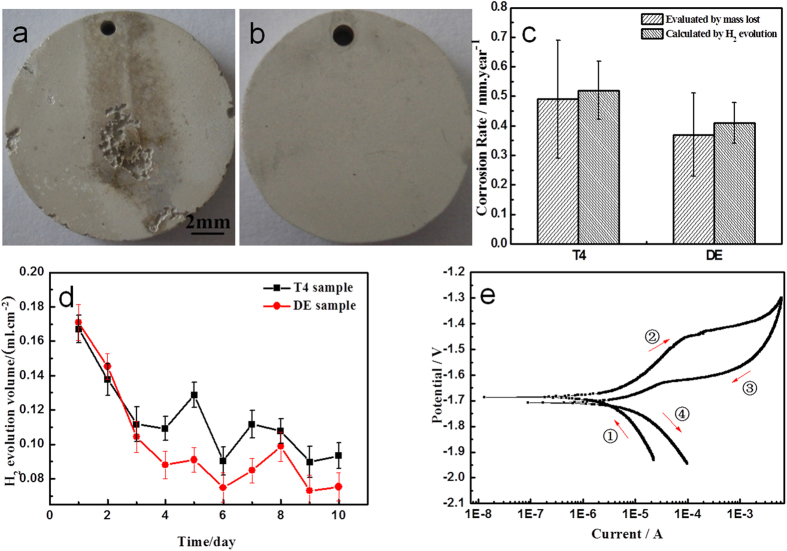
*In vitro* degradation of Mg alloy in artificial plasma. Macro corrosion surfaces of (**a**) conventional AZ31 alloy and (**b**) double extruded JDBM-2 alloy after exposing to physiological medium for 120 h and cleaning the corrosion products. (**c**) The corrosion rates of T4 and double extruded (denoted as DE) JDBM-2 alloy calculated by mass loss and H_2_ evolution. (**d**) The volume of H_2_ evolutions of T4 and double extruded JDBM-2 alloy in artificial plasma for every 1-day degradation. (**e**) Cyclic polarization curve of double extruded JDBM-2 alloy after immersed in artificial plasma for 1 hour to reach a steady state. The arrows with the sequence from one to four indicate the scanning direction of the polarization curve. Note the lower corrosion potential of the forward scan which represents the polarization behavior of the non-corroded area in the Mg alloy.

The mechanical properties of the JDBM-2 in T4, once and double extrusion conditions are shown in Table 1. After double extrusion, the ultimate tensile strength (UTS), yield strength (YS) and elongation of solution treated JDBM-2 are increased by 306%, 70 and 209% respectively (typical stress-strain curves shown in Fig. 2s). The nano particles act as barriers for the movement of dislocations during sliping and contribute to the yeild strength. On the other hand, the refined homogeneous microstructure after double extrusion is favorable for the plastic deformation. The improvement in elongation of JDBM-2 after double extrusion could be attributed to the activated non-basal dislocations in fine-grain Mg alloy with a better grain boundary compatibility effect^25^.

### *In vitro* degradation in artificial plasma

The *in vitro* degradation experiment was carried out by immersing the double extruded JDBM-2 alloy in artificial plasma for 120 h and the corrosion surface was cleaned by a standard chromium trioxide (CrO_3_) solution to remove the products, as shown in Fig. 2. Comparison was made with a conventional Mg alloy AZ31 (Fig. 2a). The corrosion of the JDBM-2 spreads across the surface, which results in very uniform and smooth corrosion morphology (Fig. 2b). In contrast, pitting corrosion is extensively observed on AZ31 Mg alloy due to the inherent micro-galvanic corrosion. The corrosion rate of the JDBM-2 (T4) measured by mass loss and hydrogen evolution is reduced by 25% from 0.49 to 0.37 mm/year after double extrusion (Fig. 2c), revealing an enhanced corrosion resistance of the JDBM-2 achieved through a fine and homogeneous microstructure. The H_2_ evolution volume (Fig. 2d) generated by the double extruded JDBM-2 is much less than that generated by the alloy in T4 condition during the investigated period. The result is found to be good agreement with the *in vitro* corrosion rate calculated by mass loss. Figure 2e shows the cyclic polarization curve for double extruded JDBM-2 after immersion in artificial plasma for 1 h. The corrosion potential of the reverse scan (−1.68 V) is greater than that of the forward scan (−1.71 V), indicating the corrosion in the corroded area on JDBM-2 alloy is likely to be suppressed by the non-corroded area, and the non-corroded area is more easily attacked by the corrosion due to its lower corrosion potential. As a result, the corrosion of JDBM-2 alloy spreads across the surface and leads to more homogeneous corrosion.

### *In vitro* cytotoxicity test

**Figure 3:**
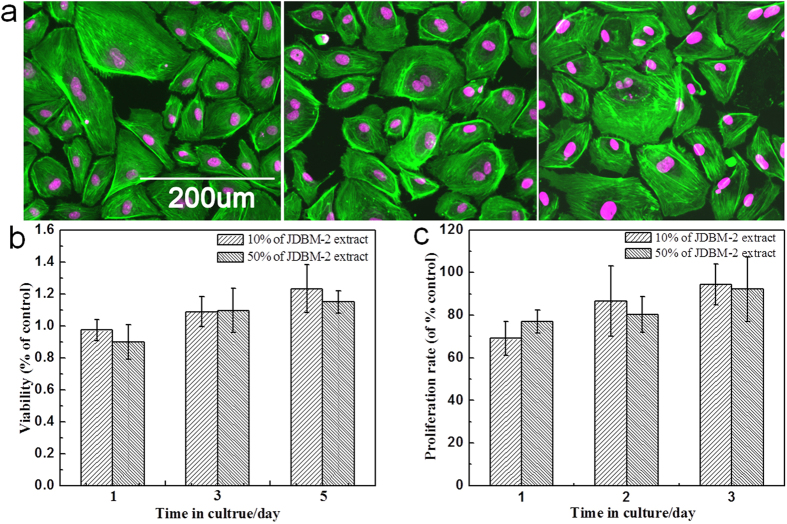
Cytocompatibility test of corrosion products *in vitro* on human endothelial cells. (**a**) Morphologies of HUVACs in negative control and Mg extract with10 and 50% dilution (from left to right) for 24 h. The cells were fixed and stained for cytoskeleton (Pholloidin: green) and nuclei (DRAQ5: purple). (**b**) HUVEC viability shown as the percentage of viable cells in the control after 1, 3 and 5 days of culture in the diluted extracts. (**c**) Proliferation ratio of HUVECs cultured in diluted Mg extracts for 1, 2, 3 days as compared to negative control.

To explore cellular response to the corrosion products, we carried out studies by *in vitro* cytotoxicity assay of HUVECs exposed to the JDBM-2 (double extrusion condition) extract. Immunofluorescence images stained for cytoskeleton and nuclei show that HUVECs appear normal and healthy with a typical morphology of cobblestone in the Mg extract compared with those cultured in normal cell culture medium (Fig. 3a). Fig. 3b illustrates HUVECs viability expressed as a percentage of negative control after 1, 3 and 5 days of culture in diluted extract media with 10 and 50% concentrations, respectively. The cytotoxicity test shows slight negative effect on HUVECs’ viability (75~99%, Grade 1) in the first day incubation, while a significant recovery of cell viability (≥100%, Grade 0) for all tests was detected on days 3 and 5. The BrdU incoporation of HUVECs (Fig. 3c) cultured in diluted JDBM-2 extracts shows slight negative effect on cell proliferation potential in the first day incubation, and then rises to more than 80% of normal cell proliferation potential for all tests with different extracts on days 2 and 3, reflecting a rapid restoration of normal cell behavior. The cytotoxicity evaluation via human endothelial cells demonstrates that the JDBM-2 extract possesses favorable biological compatible with human endothelial cell.

### *In vitro* morphology of macrophages and inflammatory secretion detection

**Figure 4:**
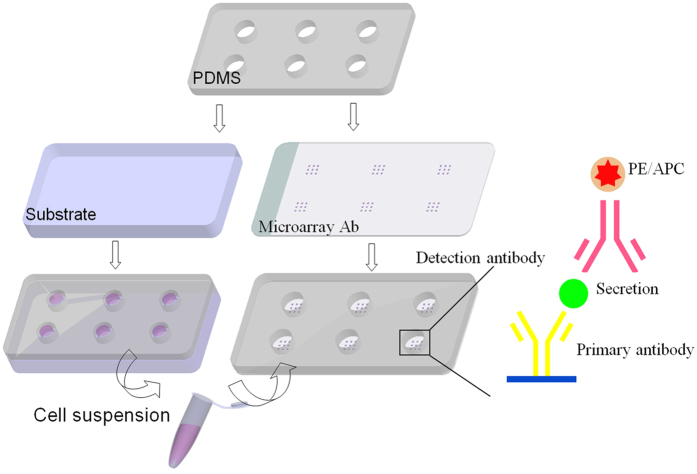
Schematic showing the experimental setup for the cell culture system and collected cytokine detection, respectively. The cell culture and cytokine detection system are fabricated from polydimethylsiloxane (PDMS) with individual hole size of Ø 7 × 5 mm on top of the substrate and microarray anti-body glass. Antibody microarrays were manufactured using a pin-spotting technique for assaying a panel of proteins produced by macrophages/FBGCs. An immunosandwich-based assay was used to detect the secretion footprint of human macrophages/FBGCs seeded on metal substrates.

**Figure 5:**
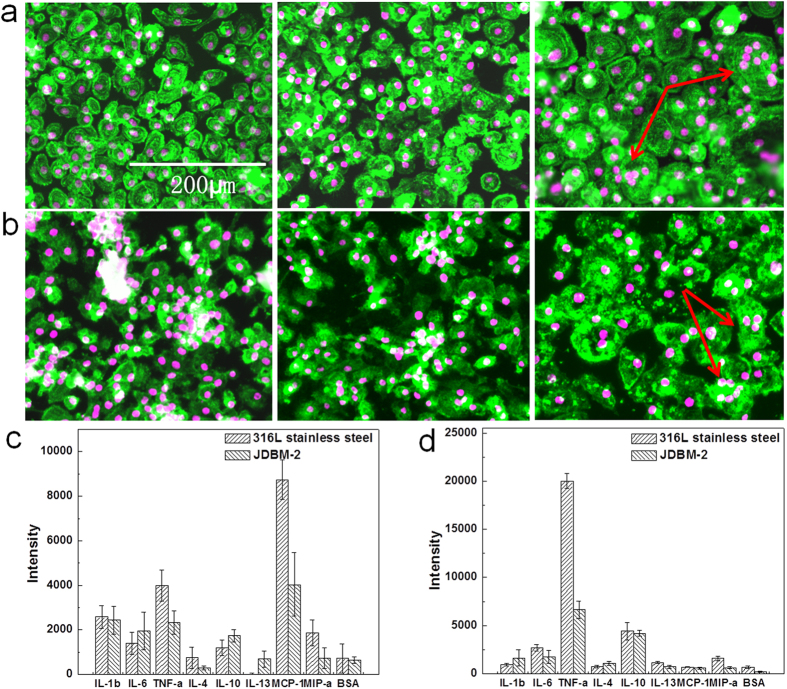
*In vitro* macrophage fusion and inflammatory cytokine and chemokine detection. Immunofluorescence images showing the morphology of macrophages/FBGCs cultured on (**a**) JDBM-2 and (**b**) 316L stainless steel for 12 h, 72 h and 144 h (from left to right). The cells were fixed and stained for cytoskeleton (Pholloidin 532 nm: green) and nuclei (DRAQ5 650 nm: purple). Multinuclear giant cells (arrowheads) were observed on both 316L stainless steel and JDBM-2 substrates due to the foreign body reaction. Cytokine and chemokine production of macrophages/FBGCs cultured on JDBM-2 and 316L stainless steel for (**c**) 24 h and (**d**) 72 h were determined by ELISA.

To investigate the effects of implanted materials on programming of macrophages into a continuum of diverse functional states, primary macrophages from human peripheral blood were cultured directly on the JDBM-2 substrate. 316L stainless steel served as negative control. A metal-based cell culture system (detailed fabrication processes and methods were illustrated in Fig. 4) was used for macrophages/FBGCs culture and supernatant collection for protein analysis. After periods of 12 h and 72 h, macrophage population distributes uniformly on 316L stainless steel while it accumulates on degradable JDBM-2 substrate, suggesting that the JDBM-2 is unfavourable for macrophage adhesion and growth and consequently leads to lower density of macrophages participating in FBR. Immunofluorescence examination shows that macrophages spread to bring their membranes into close contact, which enables macrophages to come into a cytoskeletal rearrangement and fusion-competent status for membrane merging to form multinucleated cells (Fig. 5a,b). Quantitative analysis confirms that decreased levels of macrophage fusion are driven by the JDBM-2 substrate compared with 316L stainless steel (not shown here). Results of this investigation reveal that a compromised FBR is induced by the JDBM-2. The degradation of the JDBM-2 leads to an alkalization effect with an increase in pH value of the local microenvironment, which exerts an important effect on macrophage viability^26^ and function in terms of adhesion and spread. Furthermore, the homeostasis of the surface with gradually increased corrosion products caused by the degradation of the JDBM-2 may diminish the absorption of proteins, which serve as extracellular matrix (ECM) for macrophage adhesion via several adhesion ligand-receptors interacting with those adsorbed proteins^27^. FBGC formation is a classic identifying feature of unresolved inflammation arising from the presence of implants and other foreign bodies^28^. During the foreign body reaction *in vivo*, monocytes are recruited to the site of implant insertion, and undergo maturation to macrophages which may subsequently fuse to form FBGCs. Macrophages lose most of their plasticity and mobility after they become FBGCs, at the same time, they acquire the capacity to release more effective cytokines, growth factors, and other bioactive agents to degrade foreign bodies and modulate the function of other cell types which participate in the integration and failure of implants^28^,^29^. Results of this study reveal that the temporary exist of the self-dissolved JDBM-2 stent would take advantage to avoid the long-term FBR *in vivo* after the fulfillment of its functionality.

Inflammatory cytokine and chemokine production are distinctly influenced by the surface physico-chemical properties of biomaterials (Fig. 5c,d). Tumor necrosis factor-alpha (TNF-α) is detected at lower level expressed by adherent macrophages/FBGCs cultured on the JDBM-2 possibly due to a lower cell density and less FBGC formation. TNF-α expression is consistent with the initiation of an acute inflammatory response^30^ and a key regulator to immediate tissue response by inducing other cytokines and growth factors and consequently influencing endothelial function and leukocyte transmigration^31^. Therefore these findings indicate that a compromised acute inflammatory is initiated by the JDBM-2, suggesting limited stimulation of this material for immune cells. The *in vitro* investigations enable us to isolate the interaction between biomaterials and macrophages involving in FBR and provide perspective for future *in vivo* assessments. While the investigation identifies the importance of biomaterial in modulating macrophage behavior and inflammatory process *in vitro*, the mechanisms by which macrophages preferentially accumulate and acquire a proinflammatory phenotype need to be further deciphered.

### *In vivo* angiography and IVUS results

**Figure 6:**
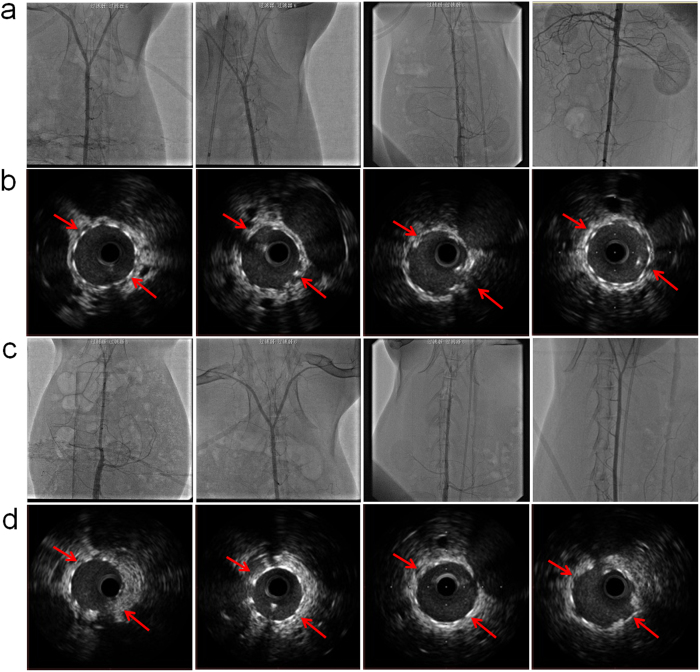
The *in vivo* aortic angiography showing no acute and late thrombogenesis as well as in-stent restenosis in the (**a**) JDBM-2 and (**c**) 316L stainless steel stent after stenting for 1 month, 2 months, 4 months and 6 months (from left to right). The corresponding follow-up IVUS images illustrating the longitudinal reconstruction of the abdominal aorta after the (**b**) JDBM-2 and (**d**) 316L stainless steel stent implantation. Note increased lumen patency and vessel size at different implantation period with nearly complete absence of neointimal hyperplasia.

We carried out *in vivo* angiography and the follow-up IVUS to investigate the safety and efficacy of the biodegradable JDBM-2 stent in rabbit abdominal aorta (Fig. 6a,b). The angiography images show no acute and late thrombogenesis, as well as serious in-stent restenosis in the JDBM-2 stents. The results are similar to those observed in the control group (316L stainless steel), as shown in Fig. 6c, demonstrating that the biodegradable JDBM-2 stent is safe and efficient *in vivo* (Fig. 6a). The follow-up IVUS was used to evaluate the expansion level, initial hyperplasia degree and the occurrences of thrombosis in different implantation periods, as shown in (Fig. 6b). The JDBM-2 stents are completely expanded and well apposed to the vessel wall with no sign of elastic recoil and fracture, reflecting excellent radial strength and compliance of the stent. In period of 1 month implantation, a thin layer of endothelium appears on the surface of the stent struts, with no significant difference compared with the results achieved in 316L stainless steel stents (Fig. 6d). The degradation period of the JDBM-2 stent in rabbit model can reach up to 6-month (arrows indicate the presence of the stent), which is consistent with the result calculated from the corrosion rate *in vitro* and proves that the JDBM-2 stent offers appropriate degradation rate *in vivo*. These results are highly striking and the major improvement in long-term mechanical durability and excellent tissue compatibility is related to the homogeneous degradation mechanism and slow corrosion rate, which makes JDBM-2 a promising Mg-based biomaterial for manufacturing biodegradable stent.

### HE staining and in stent endothelialization

**Figure 7:**
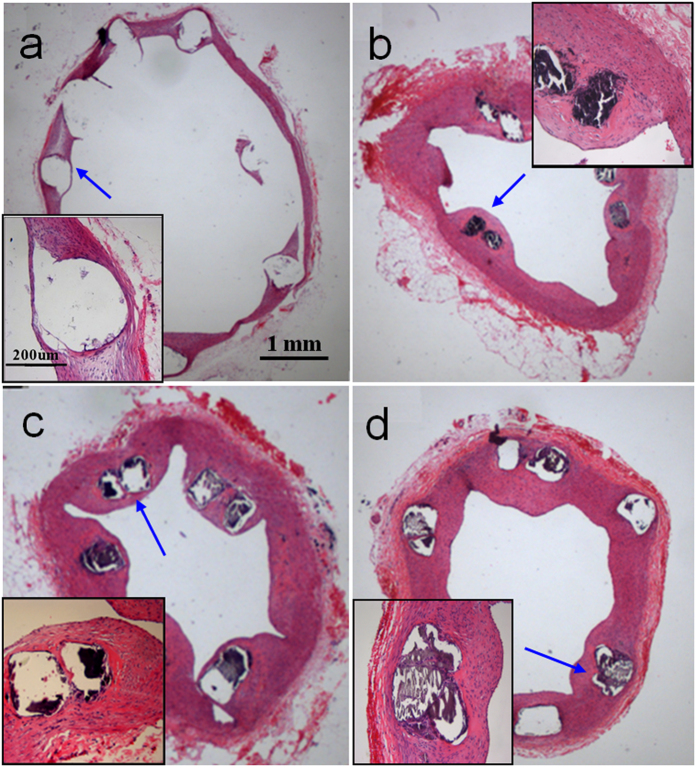
HE staining images of the stented arteries showing compromised foreign body reaction and neointimal coverage on the JDBM-2 struts in the period of (**a**) 1 month, (**b**) 2 months, (**c**) 4 months and (**d**) 6 months implantation. The inset in each image showing detailed view of the stented artery. The white and black boxes indicating that the support period of the JDBM-2 scaffold can achieve up to 6 months.

We further carried out Hematoxylin-Eosin (HE) staining to evaluate *in vivo* FBR to the biodegradable JDBM-2 stent (Fig. 7). Histologic examination reveals that a few inflammatory cells accumulate in the vicinity of the stent and a thin layer of neo-endothelium covers the surface of the struts after 1 month implantation (Fig. 7a). A continuous endothelium layer lines on the stented arteries with compromised inflammatory reaction in the extended periods of implantation. However, mild in-stent intimal hyperplasia is observed in the late periods of 4 and 6 months (Fig. 7c,d), and the inflammation basically subsides at the end of the implantation. Meanwhile, the struts of the JDBM-2 stent degrade continuously from 2 to 4 months and maintain their shapes till the end of the study. Combining the results of the *in vivo* aortic angiography and the corresponding follow-up IVUS that no early and late in-stent restenosis observed in the JDBM-2 stents, we conclude that slight inflammatory response and mild in-stent intimal hyperplasia is induced by the JDBM-2 stent during the implantation periods.

**Figure 8:**
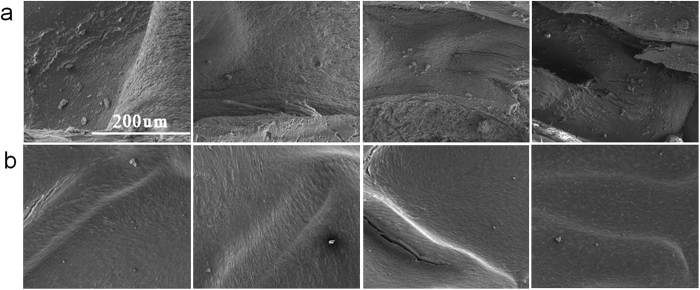
The SEM images showing neointimal coverage of the (**a**) JDBM-2 and (**b**) 316L stainless steel stent after stenting for 1 month, 2 months, 4 months and 6 months (from left to right). Comparable strut coverage is evident on the two scaffolds after 4 months implantation.

We also investigated the distal segment of the stented artery to identify in-stent endothelialization via scanning electron microscope (SEM). Results of this study indicate that complete neointimal coverage on the struts post stenting is mainly finished in the initial 4 weeks and few inflammatory cells are visible in the endothelialized stents (Fig. 8a,b). In summary, the JDBM-2 stent causes mild inflammatory reaction in the early periods, inhibits in-stent restenosis after stenting and maintains its structural and mechanical integraty till 6 months implantation. The attenuation of the accumulation of inflammatory cells and the minor inflammatory reaction in rabbit abdominal aortic arteries can be reasonably attributed to the re-endothelialization and neointimal coverage on the stent struts, which has been demonstrated as the main underlying mechanism of late stent thrombosis (ST). These findings are consistent with those of *in vitro* studies that the JDBM-2 is well compatible with endothelial cells and causes minor inflammatory response, which represents the demonstration of excellent tissue response to this stent material.

## Discussion

**Figure 9:**
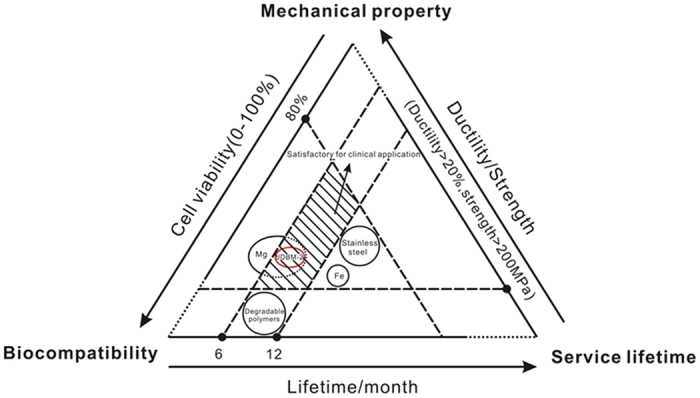
Schematic diagram of satisfactory stent material required for clinical application and limitations of current biomedical materials. The double extruded JDBM-2 alloy with excellent biocompatibility, suitable mechanical properties (yield strength: 276 ± 6 MPa, elongation: 34.3 ± 3.4%) and service life-time (up to 6-month) represents a major breakthrough to conventional stent materials and a significant advance towards the goal of successful clinical translation of full-degradable Mg-based cardiovascular stent.

A stent is a small mesh-like tubular scaffold which is mounted on a balloon and expanded inside the target coronary artery to keep the lumen open. Medical metals and polymers are the most common materials used in manufacturing cardiovascular stent over the past decades^5^,^7^,^9^,^10^,^12^,^13^,^14^,^15^. Figure 9 illustrates satisfactory stent material (shadow area) required for clinical application and limitations of current biomedical materials. The candidate materials for stent application should meet at least the three following requirements: (1) biocompatibility (cellular activity >80%): the material and its products do not pose a significant risk of toxicity to cells and must be well tolerated by the patient; (2) mechanical properties (yield strength >200 MPa, elongation >20%): the material should have sufficient strength for load-bearing application and also have acceptable ductility for a compliance match between stented and non-stented vessel areas; (3) service lifetime (up to 6–12 months): the material should stay in the diseased vessel limited to a period of 6–12 months and then can be absorbed or excreted by the surrounding tissues and the metabolic system after fulfilling its functionality. The application of biodegradable polymers and irons is limited by the relatively low mechanical strength and long corrosion period (service lifetime), respectively. While traditional stent material such as 316L stainless steel is challenged by the long-term endothelial dysfunction, chronic inflammation and delayed re-endothelialization, especially inability to adapt to growth in young patients. In this case, Mg alloys appear to be promising candidates that can potentially address the above challenges as temporary structural biomaterial for cardiovascular stent application due to the intriguing combination properties of biocompatibility, biodegradability and mechanical performance^19^,^20^,^21^,^23^,^24^,^26^. In the present study, we carried out systematic investigation with the purpose of identifying whether the double extruded JDBM-2 Mg alloy is an alternative biomaterial which is suitable for real clinical biodegradable stent application.

Stent materials with high strength and ductility allow the intraoperative mechanical deformation and a sufficient radial support to overcome the problem of elastic recoil following balloon expansion. High-strength materials also enable the design of thinner stent struts, which is beneficial for more flexible devices and lower vascular trauma^9^,^32^,^33^. As a new and effective processing technology, double extrusion was employed in this study to improve the mechanical properties of Mg alloys. The UTS, YS and elongation of solution treated (T4) JDBM-2 are increased by 306%, 70 and 209% respectively after double extrusion. Grain refinement is an effective way to strengthen mechanical properties of Mg alloys. However, long elongated grains were observed in the once extruded condition (Fig. 1b). In the basal slip system, these grains aligning along 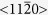 crystal direction with their c-axis perpendicular to the extrusion direction are favorably oriented to accommodate extrusion strains. While undergoing large deformation without twinning and latent hardening, once extruded Mg alloys do not have large enough stored plastic energy to trigger recrystallization, thus long elongated grains are easily formed in the microstructures of these alloys^34^,^35^. After double extrusion, the long elongated grains disappear due to the dynamic recrystallization triggered by the stored plastic energy generated in the process of hot extrusion, and the matrix grains are finer and more homogeneous, which is beneficial for the improvement of ductility. The refined homogeneous equiaxed grains achieved in complete dynamic recrystallization and the precipitation of nano particles (precipitated phases) in grain interiors and boundaries (Fig. 1d) lead to significant increase in yield strength. There are many publications on the investigation of mechanical properties of Mg alloys at room temperature. Researchers have reported remarkably high tensile yield strengths (e.g. yield strength >350 MPa) of Mg alloys while the elongation is relative low (e.g. elongation <10%)^36^,^37^,^38^. Few of them can satisfy both the high yield strength (>270 MPa) and elongation (>30%) simultaneously. In our study, the room temperature mechanical properties of the JDBM-2 alloy are fascinating. The yield strength and elongation of the JDBM-2 after double extrusion are 276 ± 6 MPa and 34.3 ± 3.4% respectively, which is close to those of clinical used medical stainless steel 316L^39^.

Corrosion is normally a drawback in engineering, however, the corrodibility of Mg alloy is of considerable interest for their application as degradable implants. Unfortunately, localized corrosion of conventional Mg alloys such as AZ31 (Fig. 2a) may lead to undesirable phenomenon such as losing efficacy or even fracture after implantation. The corrosion mechanism of Mg alloys is profoundly influenced by the second phases/precipitated phases and their distribution^40^,^41^,^42^. Mg alloys with a highly stable interface with the physiological environment would slow down the tendency of localized corrosion through reducing electron exchange between the metal and the adsorbed biological medium^43^. After double extrusion, a dense and fine dispersion of second phases/precipitated phases distributes in the JDBM-2 alloy (Fig. 1d). The slight potential difference (~25 mV) between *Mg*_*12*_*Nd* and the α-Mg matrix is anticipated to lead to increase in the tendency of interface stability in the physiological environment and consequent improvement in homogeneous degradation of the alloy^44^. Hence, we further conducted the cyclic polarization measurement to research the propensity of the double extruded JDBM-2 Mg alloy to undergo local corrosion in artificial plasma (Fig. 2e). Generally, in a cyclic polarization curve, the forward scan represents the polarization behavior of the non-corroded areas while the reverse scan is associated with the performance of the corroded areas. Due to the galvanic corrosion effect, an area with a more negative potential that acts as anode is corroded and an area with a more positive potential that acts as cathode is protected. In our study, the corrosion potential of the reverse scan in the JDBM-2 alloy is more positive than that on the forward scan (E+ < E−), which means the corrosion of the corroded area on JDBM-2 alloy is likely to be suppressed by the non-corroded area, while the non-corroded area is prone to be eroded due to a lower corrosion potential. This result can lead to reduced propensity of localized corrosion attack in the JDBM-2 alloy due to the anode and cathode switch in the galvanic corrosion process. Thus, the corrosion morphology observation (Fig. 2b) and the cyclic polarization characterization (Fig. 2e) confirm the fact that the JDBM-2 exhibits homogeneous corrosion in artificial plasma, which is desirable for biodegradable medical devices to avoid local stress concentration and enables researchers to predict the accurate degradation period *in vitro* and *in vivo*. Based on the calculation of corrosion rate, the lifetime of the JDBM-2 stent (~0.2 mm thickness) is estimated up to 6-month, which is suitable for blood vessel remodeling. Therefore, the improvement in interface stability of JDBM-2 in the physiological environment results in enhanced homogeneous corrosion tendency and potentially long-term durability *in vivo*, which can be further confirmed by the implantation of stent in animal model (Fig. 7).

The evaluation of biological responses to implants is a measurement of the magnitude and duration of the adverse alterations in homeostatic mechanisms that determine whether the medical device can be completely compatible with the surrounding tissues without causing significant damage to the vital organs. From clinical and regulatory perspective, the corrosion kinetics of Mg and its alloys exceeds that of toxic tolerance, resulting in potentially harmful perturbations to the physiological environment and a threat to neighboring tissues and organs. Mg alloy with a stable interface with the physiological environment would slow down the corrosion process, reduce the accumulation of ions and thus, improve the biocompatibility and bio-efficacy of the implant. As mentioned above, JDBM-2 with a stable interface in the physiological environment achieved through fine dispersion of second phases/precipitated phases with slight potential difference compared to the *α-Mg* matrix results in homogeneous degradation mechanism and slow corrosion rate, which is beneficial for the improvement of biocompatible outcome. For stent application, Mg alloy with a controlled degradation rate appears to have a positive effect on HUVECs deposition, proliferation and growth and potentially aids in re-endothelialization of the implant into the denuded artery *in vivo*. Enhanced endothelial cell adhesion and rapid re-endothelialization process has been suggested as a method for increasing efficacy of vascular stents through preventing thrombosis and reducing FBR^45^. At last, we performed *in vivo* assessment via implantation of JDBM-2 stent to the animal model. The result confirms excellent tissue compatibility (Figs 6, 7, 8) and up to 6-month structural and mechanical integrity of the stent *in vivo* (Figs 6,7), suggesting significant advances have been made towards the goal of successful clinical translation of biodegradable Mg-based cardiovascular stent. In summary, the double extruded JDBM-2 alloy with excellent biocompatibility, suitable mechanical properties and service life-time (Fig. 9) represents a major breakthrough to conventional stent materials and shows great potentiality for large-scale clinical application.

## Conclusions

Although Mg alloys have been investigated as biomaterials for over a decade, to the best of our knowledge, this is the first report to show that our Mg alloy Mg-2.2Nd-0.1Zn-0.4Zr (JDBM-2) with excellent cellular compatibility can integrate the mechanical properties of medical stainless steel and the degradation durability of polymer for temporary cardiovascular stent application. The yield strength and elongation of the JDBM-2 are 276 ± 6 MPa and 34.3 ± 3.4% respectively. A stable interface with the physiological environment of the JDBM-2 leads to significant improvement in homogeneous degradation mechanism and reduced corrosion rate. *In vitro* cytotoxicity test shows the JDBM-2 has negligible adverse effect on HUVECs viability and growth. This alloy also exhibits compromised FBR in comparison with 316L stainless steel via investigation of human peripheral blood derived macrophage adhesion, FBGC formation and inflammatory protein production. The implantation of the JDBM-2 stent confirms excellent tissue compatibility and up to 6-month structural and mechanical integrity *in vivo*, representing a significant step towards the goal of successful clinical translation of Mg alloys in biodegradable medical devices, and showing great potential to be alternative to conventional stent materials.

## Materials and Experiments

### Materials and heat treatments

Mg-2.25Nd-0.11Zn-0.43Zr (wt.%, JDBM-2) was cast by semi-continuous casting with high purity Mg (≥99.99%), Zn (≥99.995%), Mg-25%Nd (impurities ≤ 0.1%) and Mg-30%Zr (impurities ≤ 0.5%). The cast ingot was solution (T4) treated at 540 °C for 10 h in a protected atmosphere, quenched into water at room temperature. The ingot in T4 condition was first extruded at 290 °C with an extrusion ratio of 8 and extrusion speed of 50 mm/s for the first extrusion, followed by the second extrusion at 320 °C with an extrusion ratio of 9 and extrusion speed of 18 mm/s.

### Microstructures and Mechanical properties

Specimens (Ø12 × 5 mm) were grounded with SiC paper, mechanically polished using 3.5 and 0.5 μm diamond paste and light MgO solution, and then were etched with acid solution (10 ml acetic acid, 4.2 g picric acid, 70 ml ethanol, and 10 ml distilled water). Tensile test specimens with the dimensions of 3.5 mm width, 2 mm thickness, and 30 mm gauge length were cut parallel to the extrusion direction, polished with SiC paper and 3.5 μm diamond paste, respectively. The microstructures were observed using optical microscope (Zeiss, Oberkochen, Germany) and field emission scanning electron microscope (FE-SEM, SIRION 200, FEI, America), coupled with energy-dispersive X-ray spectrometry (EDX, INCA, Oxford, U.K). Tensile tests were carried out at room temperature on a uniaxial tensile testing machine Zwick Z100, coupled with a contact extensometer, with primary strain rate of 1 mm/min. The stress-strain curve was obtained under a uniaxial loading.

### *In vitro* degradation tests

*In vitro* degradation tests were carried out in artificial plasma (composition: 6.8 g/L NaCl, 0.4 g/L KCl, 2.2 g/L NaHCO_3_, 0.1 g/L MgSO_4_, 0.2 g/L CaCl_2_, 0.126 g/L Na_2_HPO_4_, 0.026 g/L NaH_2_PO_4_ and 1.0 g/L glucose) at 37 ± 0.5 °C for 120 h. The volume of artificial plasma was calculated based on a volume-to-sample area ratio of 40 mL/cm^2^. After immersion, the specimens were cleaned using a standard chromium trioxide (Cr_2_O_3_) solution recommended in ASTM G1-90 to remove the corrosion products. Four specimens for each group were tested, and the corrosion rate (CR) determined by mass loss was calculated using the following equation:


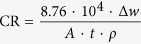


where, Δw (g) is the weight change, A(cm^2^) is the surface area of the specimen, t (h) is the immersion time and ρ (g·cm^−3^) is the density of the alloy. Cyclic polarization measurement of Mg alloy was carried out in artificial plasma by Advanced Electrochemical System (PARSTAT 2273, Princeton) with a polarization scan at a rate of 1 mV/s.

### *In vitro* cytotoxicity evaluation

Mg alloy extract was prepared using EBM (Lonza, Cat. # CC-3156) serum free medium with the surface area of extract medium ratio of 1.25 cm^2^/mL. 100 μL cell suspension with the density of 2 × 10^4^/mL was cultivated in 96-well cell culture plates in each well. After incubation for 24 h to allow attachment, the EBM medium was replaced with 100 μL of extracts with the dilution of 10 and 50% concentrations. After incubated for 1, 3, 5 days, cytotoxicity evaluation was determined using a standard MTT method. The O.D. (optical density) intensity of supernatant was measured at a wavelength of 490 nm by iMark Microplate Reader (BioTek instruments). Human endothelial cells incubated for a period of 24 h were stained with Pholloidin (Life Technologies-Invitrogen, Cat. # A22282) and DRAQ5 (Cell Signaling Technology, Cat. # 4084S) to identify cell morphologies. Proliferating endothelial cells were monitored by 5-Bromo- 20-deoxy-uridine (BrdU) incorporation into nuclei of dividing cells for 30 min, followed by BrdU Mouse mAb Labelling and Anti-Mouse IgG (H + L) Detection (Cell Signaling Technology, Cat. # 6813).

### *In vitro* macrophage assay and protein secretomic analysis

To investigate the expression of cytokine and chemokine from macrophages/FBGCs during FBR *in vitro*, human macrophages were cultured on the material surface based on a metal-based cell culture system (Scheme 1). Human monocytes were isolated from peripheral blood and differentiated into macrophages in the presence of granulocyte macrophage colony-stimulating factor (GM-CSF, Biosystems, Cat. # GMF-H4214). Macrophages adhesion and fusion were investigated by culturing cells directly on substrates with cell density of 2 × 10^5^/mL. The adherent cells were double-stained with phalloidin (Life Technologies-Invitrogen, Cat. # A22282) and DAPI (Invitrogen, cat. # D3571). Cytokine and chemokine production of macrophages/FBGCs in response to JDBM-2 and stainless steel substrates was determined by enzyme linked immunosorbent assay (ELISA).

### Stent implantation

Animal experiments were conducted under the NIH Guide for Care and Use of Laboratory Animals and approved by the Animal Ethics Committee of Zhongshan Hospital. 3–4 months old, sex unlimited, healthy and clean New Zealand white rabbits weighing 2.5–3.5 kg were brought from the laboratory animal center of Zhongshan Hosptial, Fudan University, China. JDBM-2 stent with an original dimension of Ø2 × 14 mm was fabricated in Shanghai Jiao Tong University (SJTU), China. Commercial 316L stainless steel stent was studied as control group. Serial angiography and the follow-up intravascular ultrasound (IVUS) were performed to examine the safety and efficiency of the stents. Histologic examination of stents via HE staining and SEM were used to determine the tissue response to the implant and the re-endothelialization process.

## Additional Information

**How to cite this article:** Mao, L. *et al*. A promising biodegradable magnesium alloy suitable for clinical vascular stent application. *Sci. Rep.* **7**, 46343; doi: 10.1038/srep46343 (2017).

**Publisher's note:** Springer Nature remains neutral with regard to jurisdictional claims in published maps and institutional affiliations.

## Supplementary Material

Supplementary Information Files

## Figures and Tables

**Table 1:** Mechanical properties of the JDBM-2 alloy in T4, once and double extrusion conditions.

Samples	YS (MPa)	UTS (MPa)	Elongation (%)
T4	66 ± 3	181 ± 5	10.2 ± 1.3
Once extrusion	204 ± 5	247 ± 4	20.6 ± 1.6
Double extrusion	276 ± 6	309 ± 6	34.3 ± 3.4
