# Supplementary material for: A promising biodegradable magnesium alloy suitable for clinical vascular stent application
Source: Sci Rep. 2017 Apr 11;7:46343. doi: 10.1038/srep46343 (PMC5387745; doi:10.1038/srep46343)
Supplement: Supplementary Information Files [file srep46343-s1.pdf]

A promising biodegradable magnesium alloy suitable for clinical vascular stent application

Lin Mao, Li shen, Jiahui Chen, Xiaobo Zhang, Minsuk Kwak, Yu Wu, Rong Fan, Lei Zhang, Jia Pei, Guangyin Yuan, Chengli Song, Junbo Ge & Wenjiang Ding

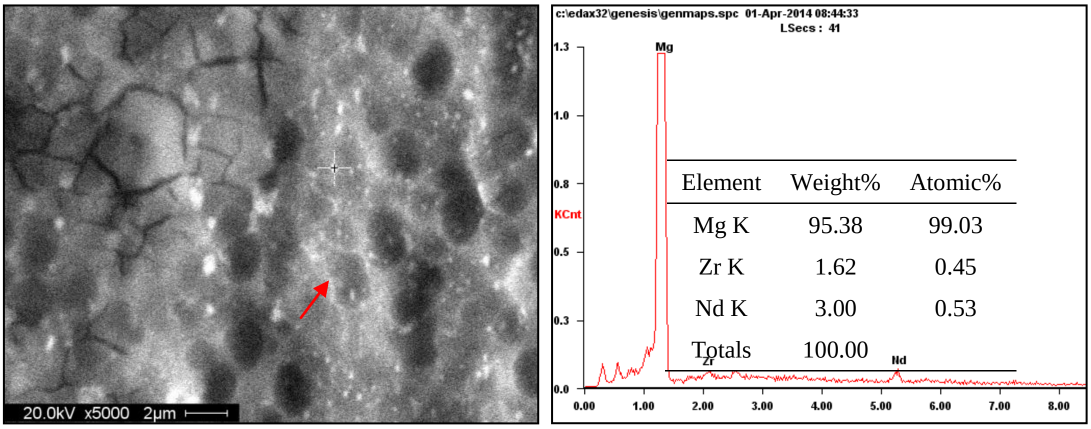

Figure 1s. EDS spectrum of the precipitate in JDBM-2 alloy. (a) Surface morphology, (b)EDS results of the precipitate (arrowhead pointing).

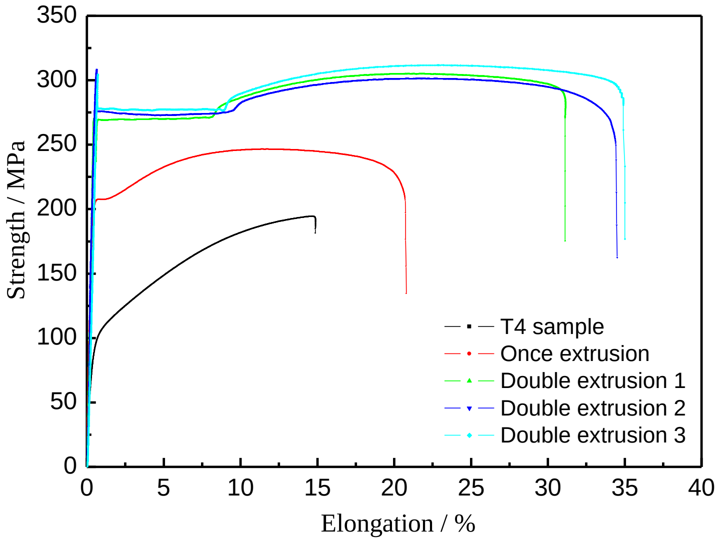

Figure 2s. Tensile curves of the JDBM-2 alloys in different conditions.
